# Accurate and efficient amino acid analysis for protein quantification using hydrophilic interaction chromatography coupled tandem mass spectrometry

**DOI:** 10.1186/s13007-019-0430-z

**Published:** 2019-05-11

**Authors:** Shrikaar Kambhampati, Jia Li, Bradley S. Evans, Doug K. Allen

**Affiliations:** 1 Donald Danforth Plant Science Center, St. Louis, MO USA; 2 United States Department of Agriculture, Agricultural Research Service, St. Louis, MO USA

**Keywords:** HILIC, Chromatography, Isobaric compounds, Amino acids, Protein hydrolysis, Protein quantification, LC–MS/MS, Soybeans, Isotopes

## Abstract

**Background:**

Methods used to quantify protein from biological samples are often inaccurate with significant variability that requires care to minimize. The errors result from losses during protein preparation and purification and false detection of interfering compounds or elements. Amino acid analysis (AAA) involves a series of chromatographic techniques that can be used to measure protein levels, avoiding some difficulties and providing specific compositional information. However, unstable derivatives, that are toxic and can be costly, incomplete reactions, inadequate chromatographic separations, and the lack of a single hydrolysis method with sufficient recovery of all amino acids hinder precise protein quantitation using AAA.

**Results:**

In this study, a hydrophilic interaction chromatography based method was used to separate all proteinogenic amino acids, including isobaric compounds leucine and isoleucine, prior to detection by multiple reaction monitoring with LC–MS/MS. Through inclusion of commercially available isotopically labeled (^13^C, ^15^N) amino acids as internal standards we adapted an isotopic dilution strategy for amino acid-based quantification of proteins. Three hydrolysis methods were tested with ubiquitin, bovine serum albumin, (BSA), and a soy protein biological reference material (SRM 3234; NIST) resulting in protein estimates that were 86–103%, 82–94%, and 90–99% accurate for the three protein samples respectively. The methane sulfonic acid hydrolysis approach provided the best recovery of labile amino acids including: cysteine, methionine and tryptophan that are challenging to accurately quantify.

**Conclusions:**

Accurate determination of protein quantity and amino acid composition in heterogeneous biological samples is non-trivial. Recent advances in chromatographic phases and LC–MS/MS based methods, along with the availability of isotopic standards can minimize difficulties in analysis and improve protein quantitation. A robust method is described for high-throughput protein quantification and amino acid compositional analysis. Since accurate measurement of protein quality and quantity are a requirement for many biological studies that relate to crop improvement or more generally, our understanding of metabolism in living systems, we envision this method will have broad applicability.

**Electronic supplementary material:**

The online version of this article (10.1186/s13007-019-0430-z) contains supplementary material, which is available to authorized users.

## Background

Proteins are ubiquitous and have enzymatic, structural and storage functions that are essential to life. Enzymes drive the metabolic reactions that sustain cellular activities in plants as well all other lifeforms. For example, Ribulose Bis-phosphate Carboxylase/Oxygenase (RuBisCO), the most abundant enzyme on the planet is integral to photosynthetic processes in plants and produces organic carbon that is needed for food, feed, fuel, fiber and as a feed stock for living organisms. Human and animal diets include significant amounts of protein and require defined levels of essential amino acids obtained predominantly from proteins derived from plants. Proteins in the form of antibodies are crucial to immune defense whereas structural proteins help define cellular and subcellular compartmentation and organization. These proteins provide physical support or function as membrane-spanning transporters that enable passage of metabolites and salts. Given the importance of proteins, methods to quantify protein are widespread; yet accurate measurement of protein and amino acids in biomass is non-trivial and relies on assumptions about composition and losses, owing to the variance in chemical and physical properties of proteins and biological matrices.

The most common methods for the quantification of total protein include: combustion-based carbon to nitrogen ratio (C/N) analysis using isotope ratio mass spectrometry (IRMS) or other elemental analysis techniques, the Kjeldahl method of titration, and biuret assays such as Lowry’s method [1], Bradford method [2] and bicinchoninic acid approach [3]. C/N-based IRMS [4] and Kjeldahl [5] analyses are sensitive and reproducible based on the extent of chemical reactions involved in tissue pyrolysis and hydrolysis, however artefacts can occur (e.g. melamine contamination or adulteration) and protein quantification requires a presumed amino acid description (i.e. Jones factor; [6, 7]). Spectrophotometric and colorimetric approaches can be used for determining protein levels but are subject to interference from other compounds [8–11]. Thus, spectrophotometry is limited to detection and quantification of soluble protein in conditions compatible with the respective assay and is based on strategies for protein purification that are widespread [9]. Some protein will be lost as a result of purification, so whether the goal is to measure total or a specific protein is important to establish prior to choosing a method. In addition, the amino acid composition and sequence affect the accuracy of spectrophotometric methods, thus a primary application is the quantification of a known pure protein relative to a measured standard or relative comparison between like samples. Importantly, none of the described methods measure amino acid composition.

Amino acid compositional analysis (AAA) can be used to assess protein levels (i.e. when proteins are hydrolyzed) or to identify proteins as a complementary approach to peptide mass finger printing or MS/MS sequencing [12]. The quality of protein is determined by the amino acid composition and hence AAA can guide plant breeding and engineering efforts to enhance food, feed, nutraceutical and pharmaceutical applications. Amino acids with or without derivatization are separated utilizing, (ultra) high performance liquid chromatography (UHPLC/UPLC or HPLC)-, gas chromatography (GC) or capillary electrophoresis (CE) prior to detection by absorbance, fluorescence, or mass spectrometry (MS). As an example, when primary amines react with fluoraldhyde o-phthaldialdehyde (OPA), a derivatizing reagent, isoindoles are formed and contain a fluorophore excited at 302–395 nm and detected at 420–650 nm [13]. Fluorescence-based detection can achieve limits < 1 pmol [13–16]; but the derivatives have limited stability [17] and measurement can be additionally compromised by interfering signals from naturally fluorescing compounds. MS linked to GC (i.e. GC–MS) can circumvent the latter but routine derivatizations such as silylation [18] that enhances compound volatility also reduces stability [19, 20], and may degrade amino acids or produce multiple reaction products that complicate analyses. In addition, the derivatized standards must be made fresh prior to each analysis.

Liquid chromatography does not require derivatizations to enhance volatility and when coupled with tandem mass spectrometry (LC–MS/MS) provides a burgeoning set of hyphenated techniques to complement polar compound analysis. LC–MS/MS has high selectivity and is sensitive to detection of both derivatized [21] and underivatized amino acids [22, 23]; however, the coupling of LC with MS is restricted to compatible volatile solvents unless additional desalting processes are introduced. LC–MS/MS has enabled rapid throughput with reverse phase columns to resolve most amino acids [24]. Ion pair reagents [25, 26] further improve the resolution but contaminate the MS system, resulting in suppressed signals and affecting sensitivity for other applications.

Hydrophilic interaction liquid chromatography (HILIC) columns can potentially avoid these difficulties. HILIC columns partition contents between a mobile phase that is significantly hydrophobic with a stationary phase that is sufficiently hydrophilic to retain partially water-soluble compounds [27]. The retention of polar compounds aids separation [28–30], and in some cases has enabled the resolution of isobaric compounds [31, 32]. Recently, HILIC based approaches have been used for successful resolution of both proteinogenic [31, 33–36] and non-proteinogenic [37–39] amino acids for animal and plant matrices. By pairing with tandem mass spectrometry, HILIC could be used to sensitively detect and quantify protein content without the need for chromophore additions or other derivatizations and would enable resolution of all amino acid isotopologues when metabolic applications with isotopic labeling are of interest. Specifically, the masses of labeled (1) asparagine, aspartate, leucine, and isoleucine, (2) valine, threonine, proline, and cysteine and (3) glutamate, glutamine, lysine and methionine overlap, therefore chromatographic separation are necessary to avoid ambiguous results.

In this study, a HILIC-tandem MS approach was used to assess free amino acids and tested with multiple hydrolysis techniques to quantify protein level and composition by an isotope dilution strategy. All twenty amino acids were quantified using commercially available isotope labeled internal standards within a linear range of four orders of magnitude that accurately accounts for losses in signal intensities, interference from the biological matrix, and degradation that otherwise are problematic [25, 40]. Recovery of labile amino acids including cysteine, methionine and tryptophan is particularly germane because these are frequently the focus of efforts to improve nutrition. A ^13^C, ^15^N uniformly labeled amino acid standard mixture that contained all twenty proteinogenic amino acids as internal standards was spiked into samples for absolute quantification and to assess amino acid degradation and losses during sample preparation. The method includes sample preparation, chromatographic resolution and mass spectrometric detection of amino acids and does not require costly, toxic, and sometimes proprietary chemical derivatization reagents that produce unstable products, give variable reaction efficiencies and require significant sample preparation time that reduces throughput. The MS approach avoids ambiguity because quasi-molecular ion masses (mass-to-charge ratios also could be used) not chromophores are detected. Thus, small retention time drifts do not require additional external standard runs. The studies indicate that hydrolysis is complete (86–103%) and that protein can be accurately quantified with the prescribed isotopic dilution-based analysis without the need for additional experiments to assess more labile amino acids as in other AAA methods.

## Methods

### Reagents and materials

^13^C, ^15^N isotopically labeled amino acid standards were obtained as a cell free amino acid mixture (Sigma-Aldrich, St. Louis, Missouri, USA). Amino acid standard curves were generated using Thermo Scientific™ Pierce™ Amino acid standard H (Fisher Scientific, Fair Lawn, New Jersey, USA) with the addition of tryptophan (Sigma-Aldrich, St. Louis, Missouri, USA) HPLC grade acetonitrile was purchased from Fisher Bioreagents™ (Fisher Scientific, Fair Lawn, New Jersey, USA). Ammonium formate (99%), obtained from Acros Organics (New Jersey) was dissolved and combined with ultrapure water and HPLC grade acetonitrile (Fisher Scientific, Fair Lawn, New Jersey, USA) to make solvents A and B and then filtered through 0.2 µm Durapore^®^ membrane filters (Millipore-Sigma, Burlington, Massachusetts, USA) prior to use. Ubiquitin and bovine serum albumin protein standards were obtained from Sigma-Aldrich (St. Louis, Missouri, USA). Hydrolysis reagents including: 6 M HCl (24308), 4 M methane sulfonic acid solution with 0.2% tryptamine (w/v) (M4141) and hydrogen peroxide (16911) were from Sigma-Aldrich (St. Louis, Missouri, USA). Soy flour standard reference material^®^ 3234, was purchased from National Institute of Standards and Technology (NIST, Gaithersburg, Maryland, USA). The hydrolysis/derivatization vial used for all hydrolyses procedures were a Kimble^®^ product (Vineland, New Jersey, Part # 896820).

### HPLC–MS/MS instrumentation, chromatographic and MS parameters

All samples were separated and analyzed using a Shimadzu Prominence-xR UFLC (UPLC) system connected to a SCIEX hybrid triple quadrupole-linear ion trap MS equipped with Turbo V™ electrospray ionization (ESI) source. Positive ion mode was used for all amino acids, except cysteic acid which was ionized in negative mode. A 3 µl sample was injected on the Infinity Lab Poroshell 120 Z-HILIC column (2.7 µm, 100 × 2.1 mm; Agilent Technologies, Santa Clara, CA, USA) and amino acids were eluted with an increasing gradient of 20 mM ammonium formate in water (A) and acetonitrile: water (90:10) at a final concentration of 20 mM ammonium formate (B), pH 3.0 for both solvents. A constant flow of 0.25 mL/min was provided to separate amino acids through gradient elution, of 100–90% B over 2 min and then to 50% B over the next 6 min followed by returning to 100% B over 30 s before re-equilibrating the column for 6.5 min. Electrospray ionization source conditions included: ion spray voltage, 4.5 kV (ESI+ and ESI−); ion source temperature, 400 °C; source gas 1, 45; source gas 2, 40; and curtain gas, 35. Ions were detected and monitored using a targeted MRM approach with the parameters listed in Additional file 1: Table S1 based on direct injections of individual amino acid standards. Data were analyzed using the quantitation wizard available in Analyst (v. 1.6.2) software (SCIEX, Concord, Canada). Amino acid concentrations and losses were calculated from direct comparison of analyte peak areas relative to isotope labeled internal standards.

### Comparison of protein hydrolysis techniques

Seven hydrolysis methods were initially compared using 6 M HCl or 4 M methane sulfonic acid with a combination of oxidizing agents (hydrogen peroxide, performic acid) and antioxidants (beta-mercaptoethanol (β-ME), phenol, tryptamine) (Additional file 2: Figure S1) [41–47]. All hydrolyses were performed in a hydrolysis/derivatization vial (Kimble^®^, Vineland, NJ) that was purged with nitrogen and evacuated prior to heating. From the initial studies a subset of three approaches were further characterized. In the first method, hydrogen peroxide (H_2_O_2_; 30% v/v) was used to oxidize susceptible amino acids at 30 °C for 30 min followed by vapor phase hydrolysis with 6 M HCl (24 h at 110 °C, 0.4% β-ME, v/v). A second approach included hydrolysis without oxidation and the third strategy included 4M methane sulfonic acid containing 0.2% tryptamine (110 °C for 22 h). Protein standards were hydrolyzed in triplicate in 350 µl flat bottom glass inserts tubes within the vacuum chamber. At the end of the hydrolysis, samples were brought to room temperature, neutralized with 4 M NaOH, and dried in a speed vacuum centrifuge. Ultrapure water was used to solubilize amino acids prior to filtering (0.45 µm cellulose acetate centrifuge filters, costar^®^, Corning Inc.) and storing at − 80 °C until use. Protein quantification by AAA was assessed relative to weighed values and known compositions. Soy flour standard reference material^®^ 3234, obtained from NIST was compared to the certificate of analysis (CoA) [48].

### Derivatization with AccQ-Tag and UPLC based detection

AccQ-Tag derivatization, detection, and quantification were carried out using the manufacturer’s protocol. In brief, the 6-aminoquinolyl-N-hydroxysuccinimidyl carbamate (AQC) reagent was reconstituted in 1 mL acetonitrile and heated at 55 °C until dissolved completely. Aliquots were combined with AccQFlour borate buffer and reconstituted AQC reagent, incubated at room temperature then heated to 55 °C for 10 min prior to transferring into amber glass vials for HPLC injection.

Sample analysis included a Waters UPLC system with a UPLC Binary Solvent Manager and Sample Manager equipped with a Cortecs UPLC C18 (1.7 µm, 2.1 × 100 mm) column. The solvents used were the manufacturer-supplied AccQ-Tag Ultra Eluent A and AccQ-Tag Ultra Eluent B. The binary gradient involved a brief hold at 0.1% for 54 s followed by increasing %B to 9.1 over 5.74 min and then to 21.2 until 7.74 min. The column was then flushed by increasing the %B to 59.6 by 8.04 min and holding until 8.64 min. The %B contribution was then reduced to 0.1 by 8.73 min and the column equilibrated for 0.77 min. Amino acids were detected by photodiode array and quantification based on standard curves generated with Thermo Scientific™ Pierce™ amino acid standard H (Fisher Scientific, Fair Lawn, New Jersey, USA) including the addition of tryptophan, cysteic acid and methionine sulfoxide standards at known levels (Sigma-Aldrich, St. Louis, Missouri, USA).

## Results and discussion

### Proteinogenic amino acids can be resolved using HILIC

**Fig. 1:**
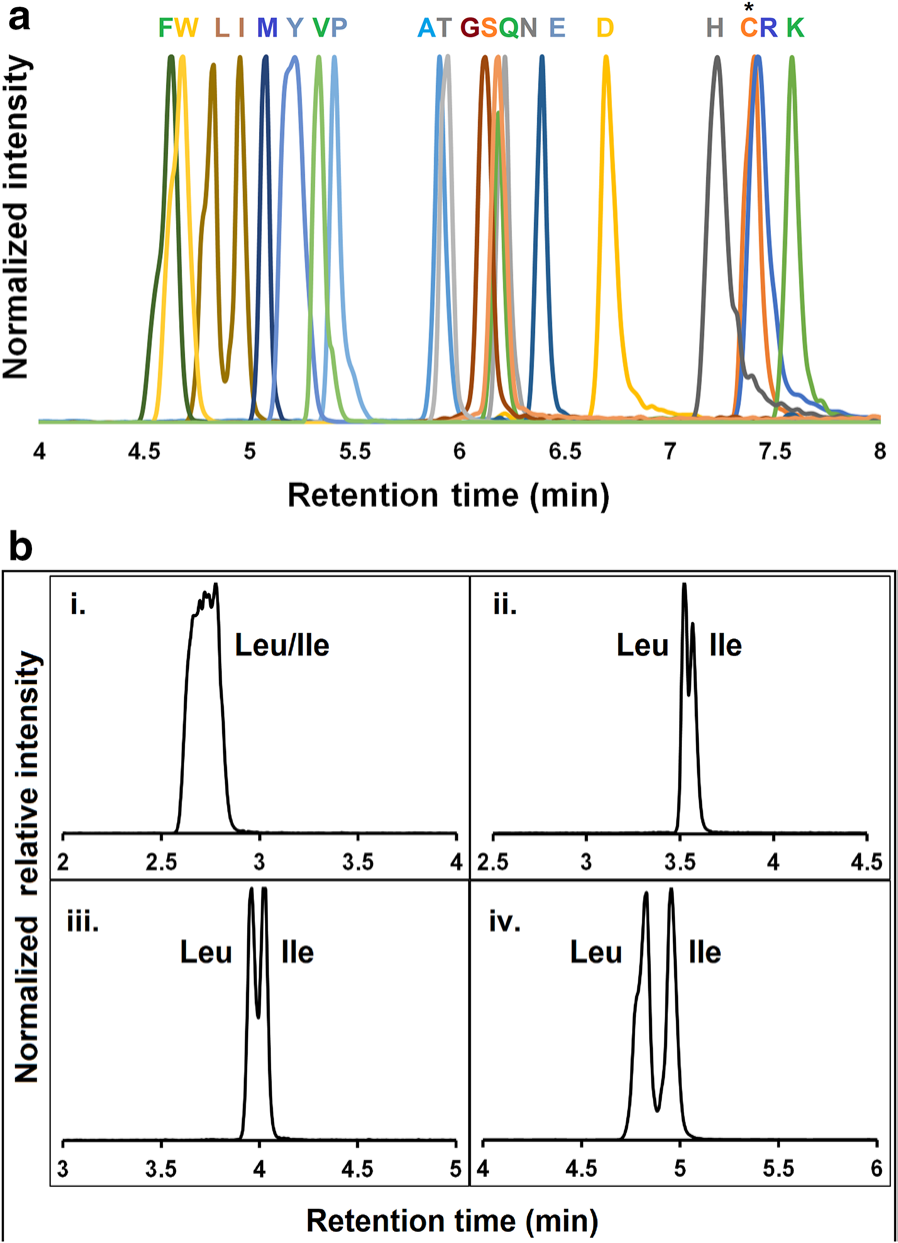
**a** Chromatographic separation of twenty amino acids using a Z-HILIC column **b**. Leucine and isoleucine were separated capitalizing on the HILIC-water layer. (i) Leucine and isoleucine are not resolved with a linear gradient 10–50% solvent A over eight minutes. (ii–iv) Represent increased separation of these compounds with changes in gradient over the first 2 min (ii 0–25% solvent A, iii 0–15% solvent A, iv 0–10% solvent A). *Cysteine was detected as cystine. Ile was quantified using a distinct mass trace (Additional file 1: supplementary Table 1)

Protein determination from complex biomass samples is challenging and requires preparative methods that are not biased by differences in protein solubility. In addition, accurate quantification of protein requires knowledge of the amino acid composition which therefore must be determined for each sample. To address the latter, a 15-min LC method was developed with a binary gradient composed of 20 mM ammonium formate in water (A) and 20 mM ammonium formate in acetonitrile: water (90:10) (B) adjusted to pH 3.0. The 20 proteogenic amino acids (Fig. 1a) were resolved chromatographically; through a solvent gradient profile that included initiating runs with 100% solvent B (0.25 mL/min), transitioning to 10% solvent A within 2 min, then to 50% solvent A over 6 min. Amino acids were eluted within this period and the initial conditions were re-established within 30 s followed by equilibration for 6.5 min. Chromatographic resolution of leucine (Leu) and isoleucine (Ile), that are isobaric and challenging to separate, was used to benchmark the method. The water partitioning capacity of HILIC columns included 0–10% solvent A to separate the isobars [Fig. 1b(i–iv)]. Unlike reverse phase chromatography methods paired with modifiers [21, 24–26], this approach can be adapted to existing liquid chromatography systems without requiring extensive instrument clean-up time.

### Quantitative amino acid signal responses extend over four orders of magnitude using ^15^N ^13^C labeled internal standards

Eluted amino acids were ionized by an electrospray approach (ESI) and detected using a multiple reaction monitoring (MRM) method (parameters described in Additional file 1: Table S1). Amino acids were monitored as positive ions except for the oxidized form of cysteine, cysteic acid, which was detected in negative ion mode. Peak areas of an equimolar unlabeled standard mix were integrated and normalized to that of the largest peak, phenylalanine, to obtain relative response factors (Table 1). The differences in matrix effects and ion suppression for individual amino acids preclude the use of a single internal standard, such as norvaline [15, 49, 50] thus a commercially available mix of uniformly ^13^C, ^15^N labeled amino acids was used as internal standards enabling an isotopic dilution strategy. When labeled internal standards are provided the changes in instrument performance and operation, sensitivity, influences of sample matrix, and losses during sample preparation are accounted for such that the ratio of analyte to internal standard is maintained and the amount of a compound can be quantified accurately without continually regenerating external calibration curves. A serial dilution of an unlabeled amino acid standard mixture (0.1–1000 pmol) spiked with a constant amount of internal standard mix (20 pmol) was prepared and the ratio of analyte to internal standard used to generate a standard curve for each of the amino acids (Additional file 3: Figure S2). Then the linear range, limits of detection (LOD) and quantification (LOQ) were determined for each amino acid as described in Table 1.

**Table 1:** Performance characteristics of 20 amino acids for quantitative analysis

	Retention time	^a^RRF	^b^LOD (pmol)	^c^LOQ (pmol)	Min (pmol)^d^	Max (pmol)^d^
Alanine	5.90	0.59	11.62	35.21	0.50	1000
Arginine	7.41	42.39	0.83	2.53	0.10	1000
Asparagine	6.22	3.71	5.55	16.81	0.20	1000
Aspartate	6.69	4.66	7.76	23.52	0.30	1000
Cystine	7.38	5.83	0.20	0.61	0.15	750
Glutamine	6.18	1.39	0.04	0.12	0.03	150
Glutamate	6.39	9.42	0.44	1.32	0.10	1000
Glycine	6.11	0.34	8.42	25.53	2.50	1000
Histidine	7.22	51.16	1.18	3.58	0.10	1000
Isoleucine	4.95	9.87	0.14	0.42	0.10	1000
Leucine	4.83	57.03	1.73	5.23	0.10	1000
Lysine	7.58	24.58	2.55	7.72	0.50	1000
Methionine	5.08	10.77	0.10	0.30	0.10	1000
Phenylalanine	4.63	100.00	1.15	3.49	0.10	1000
Proline	5.40	96.77	0.78	2.36	0.10	1000
Serine	6.18	3.91	9.24	28.01	2.50	1000
Threonine	5.94	6.31	5.83	17.68	0.50	1000
Tryptophan	4.68	42.97	1.03	3.13	0.10	1000
Tyrosine	5.24	9.42	1.29	3.90	0.10	1000
Valine	5.33	28.45	0.49	1.48	0.10	1000

^a^RRF represents response factors relative to phenylalanine which showed a maximum response. ${\text{RRF}} = 100*\left( {Peak\;Area\frac{analyte}{Phenylalanine}} \right)$

^b^LOD = 3.3 × σ/slope of the standard curve

^c^LOQ = 10 × σ/slope of the standard curve

^d^ range of concentrations examined

(σ = SD of peak areas of least detectable concentration, *n* = 3)

Fifteen of the twenty proteinogenic amino acids had LODs less than 3 pmol injected on column. Glycine, alanine and serine had higher LODs of 8.42, 11.62 and 9.24 pmol respectively (Table 1) that reflect ionization efficiencies and possibly signal suppression from matrix, solvent, or salt clusters. Despite the differences, the isotope dilution strategy produced reproducible results because the factors contributing to variability affected the amino acids in unlabeled samples as well as the internal standards.

### Proteins can be accurately quantified using multiple hydrolysis methods with internal labeled standards

Protein hydrolysis efficiency and processing losses were assessed by quantifying a known amount of a protein standard. A preliminary hydrolysis test was performed using seven different methods described in literature [41–47] based on a combination of reaction conditions, with agents to promote or diminish oxidative reactions that maximize sensitive amino acid detection (Additional file 2: Figure S1). Three methods that gave maximal recovery and had low standard errors for most of the amino acids were further pursued. Detection of the most labile amino acids is presented in Additional file 2: Figure S1. As described in the methods, hydrolysis with either 6 M HCl or 4 M methane sulfonic acid was performed in combination with hydrogen peroxide (H_2_O_2_) to induce oxidation or 0.4% β-ME or 0.2% tryptamine as anti-oxidants for reliable peak quantitation. The AAA and protein hydrolysis approaches were evaluated using BSA and ubiquitin standards.

The protein standards were greater than 98% pure (vendor supplied quality control values) therefore, comparison of a known amount of protein with that calculated after hydrolysis and LC–MS/MS analysis provided a strategy to accurately assess protein quantification. Aliquots of labeled amino acid standards were included with each hydrolysis to control for individual losses during the process. Each amino acid was quantified through the ratio of analyte peak area relative to the equivalent isotopically labeled (^13^C, ^15^N) internal standard. Total protein concentration was calculated as the sum of individual amounts of amino acids. The amount of two protein standards was 86–103% of the known value for ubiquitin and 82–94% for BSA depending on the hydrolysis approach (Table 2).

**Table 2:** Quantitation of ubiquitin and BSA standards as determined by LC–MS/MS isotope dilution and AccQ-Tag methods

	H_2_O_2_ + 6 M HCl + β-ME	6 M HCl + β-ME	4 M MetS + 0.2% Tryptamine
% Quantitation of actual* value using LC–MS/MS			
Ubiquitin	86.7 ± 8.5	100.8 ± 0.3	103.0 ± 0.7
BSA	82.4 ± 0.8	92.7 ± 1.3	94.6 ± 0.5

	H_2_O_2_ + 6 M HCl + β-ME	6 M HCl + β-ME	4 M MetS + 0.2% Tryptamine
% Quantitation of actual* value using AccQ-Tag			
Ubiquitin	79.6 ± 9.7	88.9 ± 0.5	48.8 ± 9.4
BSA	80.5 ± 1.1	59.3 ± 7.9	14.6 ± 5.1

Variability is expressed as standard error of mean (*n* = 3)

H_2_*O*_*2*_ hydrogen peroxide, *HCl* hydrochloric acid, *β*-*ME* beta-mercaptoethanol, *MetS* methane sulfonic acid

*Actual values are based on weighed amount of standards

**Fig. 2:**
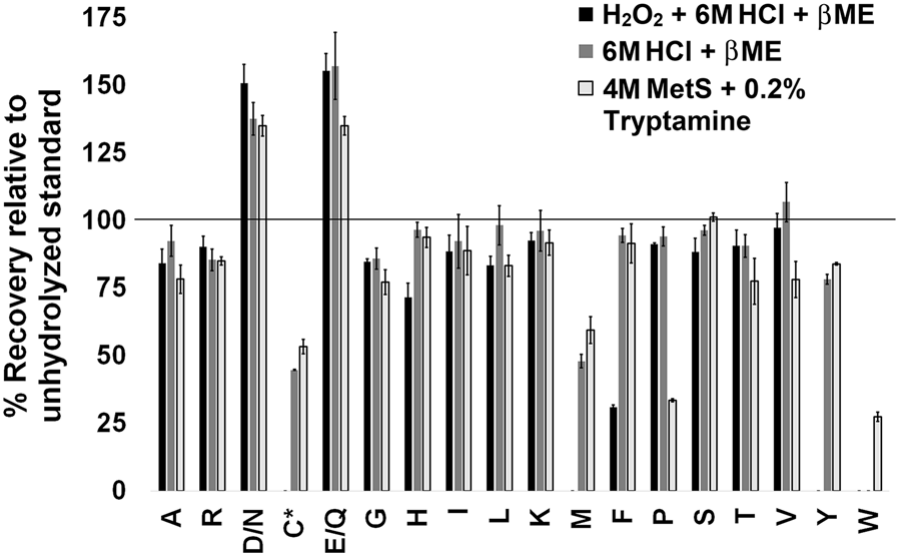
Quantitation of individual amino acids upon hydrolysis using three different methods. ^13^C, ^15^N labeled amino acid standards were hydrolyzed and compared to an unhydrolyzed standard. *Cysteine was detected as cystine. D/N represents the combination of aspartate and asparagine detected as aspartate, E/Q represents the combination of glutamate and glutamine detected as glutamate

Though accounted for through the isotopic dilution strategy, losses for individual amino acids were determined by comparing peak areas of isotopically labeled standards with or without prior hydrolysis (Fig. 2). Since the amino acid standards do not require hydrolysis the comparison indicates the extent of amino acid degradation during the process. When quantitative hydrolysis is desired [15, 46, 47] the time needed to break all peptidyl bonds can result in additional side chemical reactions that alter or degrade amino acids. Single point calibrants that normalize for losses in signal intensity such as: norvaline, norleucine, alpha-amino butyric acid or sarcosine have distinct responses and therefore cannot account for the differences in specific amino acids [15]. Standard addition methods (i.e. spiking in known amounts of each standard) can account for differences in response but require twice the number of samples and increase time and cost [49, 50].

Comparison of standards with and without hydrolysis indicated significant degradation of a small number of amino acids including cysteine, methionine and tryptophan [41–45]. Other amino acids had losses of approximately 25% or less in most cases (Fig. 2). Asparagine and aspartate, glutamine and glutamate were analyzed together because asparagine and glutamine are deamidated during the hydrolysis process. Deamidation of asparagine and glutamine to the corresponding carboxylic acids does not significantly impact quantification of total protein because the molecular masses are near equivalent (i.e. asparagine is 132 g/mol, aspartate is 133 g/mol, glutamine is 146 g/mol and glutamate is 147 g/mol). Cysteine (Cys), methionine (Met) and tryptophan (Trp) are susceptible to oxidative degradation during hydrolysis resulting in variable recovery. These three amino acids are important targets for bioengineering and crop improvement, due to their relative low content in soybean protein despite being a rich source of other essential amino acids [51]; therefore, quantification of the labile amino acids was scrutinized during the method development. Hydrolysis with 6 M HCl and 4 M methane sulfonic acid resulted in 45 and 53% of Cys and 48 and 60% of Met respectively without producing significant cysteic acid and methionine sulfoxide. When protein was treated with H_2_O_2_ prior to hydrolysis the stable oxidized forms were generated. Trp was completely degraded in both methods that used 6 M HCl for hydrolysis, [52], while the methane sulfonic acid method, with 0.2% tryptamine resulted in a 27% recovery (Fig. 2) qualitatively similar to prior reports [53]. Phenylalanine losses were significant in 6 M HCl method that was subject to prior oxidation compared to the other two methods, and tyrosine (Tyr) was not detected, likely due to halogenation [54]. Proline losses due to processing and degradation were also significant, however even when present in significantly reduced quantities the amount of amino acids could be accurately assessed through the isotopic dilution strategy (Tables 2, 3).

**Table 3:** Calculated amounts of labile amino acids, after different hydrolyses, represented in percentage of total protein, BSA

Analyte	Expected (%)	Observed (%, LC–MS/MS)			Observed (%, AccQ-Tag)		
		H_2_O_2_ + 6 M HCl + β-ME	6 M HCl + β-ME	4 M MetS + 0.2% tryptamine	H_2_O_2_ + 6 M HCl + β-ME	6 M HCl + β-ME	4 M MetS + 0.2% tryptamine
Cys*	5.77	5.3 ± 0.51	4.23 ± 0.32	4.56 ± 0.34	5.38 ± 0.10	1.11 ± 0.06	1.75 ± 0.32
Met*	0.82	ND	0.79 ± 0.01	0.76 ± 0.01	1.52 ± 0.02	0.29 ± 0.01	0.46 ± 0.02
Trp	0.49	ND	ND	0.59 ± 0.02	0.04 ± 0.00	0.05 ± 0.00	0.23 ± 0.01

Variability is expressed as standard error of mean (*n* = 3). *ND*, not detected

*Cysteine was measured as cysteic acid in hydrolysis that involved prior oxidation and as cystine otherwise. Similarly, methionine was measured as methionine sulfoxide upon oxidation and as methionine in the two methods that did not involve prior oxidation

### AccQ-Tag based protein quantitation requires parallel processing and multiple runs for accurate results

For comparison, proteins were also quantified using a commercially available UPLC system (AccQ-Tag) based on AQC derivatives detected by photodiode array (PDA). When the protein quantity was calculated by the sum of all amino acid amounts, the results were variable and significantly underestimated the total protein level (i.e. 48–90%, and 15–81% for ubiquitin and BSA respectively) depending on the hydrolysis approach (Table 2).

The low protein estimates from the AccQ-Tag method reflect losses that could not be accounted for during hydrolysis and processing. The AccQ-Tag method is typically used to establish relative amino acid composition and involves several preparations (using different hydrolysis approaches in parallel) for each sample to quantify the maximum number of amino acids. Accounting for amino acid losses is not possible, therefore if one preparation is used to enhance throughput the approach will be more apt to underestimate total protein level.

**Fig. 3:**
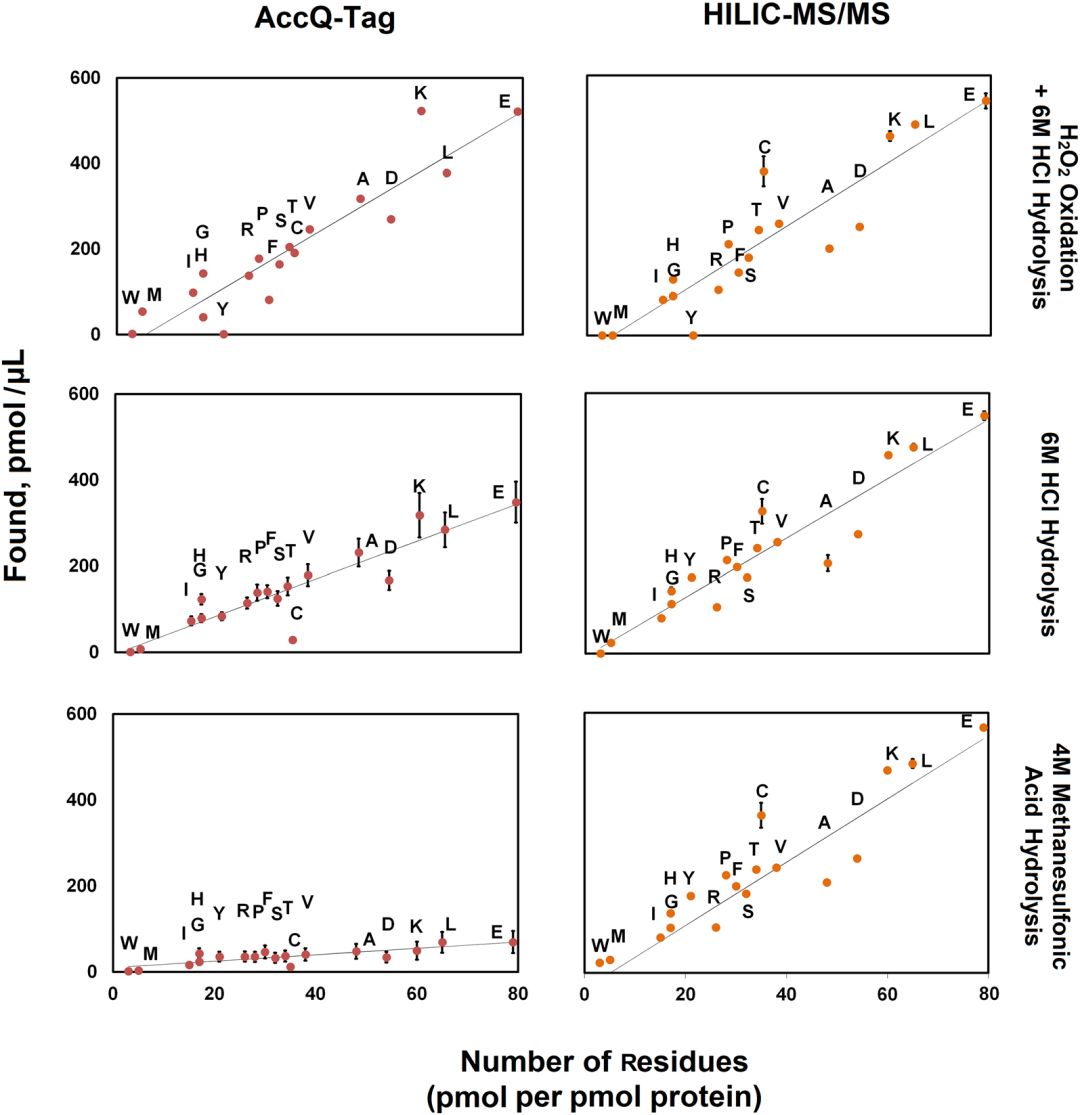
Quantitative comparison of amino acids using AccQ-Tag and HILIC-MS/MS. Individual amino acids concentrations (y-axis) as a function of hydrolysis technique and the number of residues present in BSA. The trendline represents the expected amounts of individual amino acids for protein recovered. Values that are above the trendline represent overestimation and below trendline represents underestimation of specific amino acids

Alternatively, when assessing a standard or pure protein with known sequence, the measured concentration of all or a subset of amino acids relative to the number of residues can be plotted and the regressed line of best fit used to establish the protein level [55]. Since this latter approach relies on knowledge of the sequence of the protein(s) or mole fractions of the amino acids present in the sample, it is not applicable to complex protein mixtures as in most biological samples. BSA that was hydrolyzed by different methods was also analyzed using the regression approach with measured amino acid amounts established from AccQ-Tag and HILIC-MS strategies (Fig. 3). Hydrolysis with HCl including oxidation by H_2_O_2_ (Fig. 3a) resulted in similar best fit lines for AccQ-Tag and HILIC-MS strategies indicating that the amino acid amounts determined from both methods were qualitatively similar and consistent with the values of total protein based on summed amino acid levels in Table 2 (i.e. 80%, AccQ-Tag; 82% HILIC-MS for BSA). Possibly if a subset of most reliable amino acids were chosen for quantification the methods would result in a similar level of total protein that approached 100% from either method. However, when other hydrolysis techniques were considered, the HILIC-MS-based isotope dilution approach improved with better accounting for losses that resulted in protein estimates which were 92–95% accurate; providing a superior strategy to assess protein with unknown amino acid composition (Table 2, Fig. 3).

### Quantitative recovery of soybean protein in defatted flour

Defatted soybean meal (soy flour), a complex biological sample that is a common source for food and feed protein applications, was obtained as a reference material (SRM-3234) from the National Institute of Standards and Technology (NIST). 16 out of 18 amino acids were quantified to similar levels regardless of the hydrolysis approach (Table 4). Absence of Trp indicated interference from carbohydrates during hydrolysis [42, 52] which can be removed through prior processing [56–58]. When H_2_O_2_ was added to oxidize methionine and cysteine, subsequent hydrolysis with 6 M HCl resulted in halogenation and loss of tyrosine [54]. The total amount of protein from the three hydrolysis methods was 90, 94 or 100% of the reported NIST value (Table 4) with the latter based on a combination of Kjeldahl, thermal conductivity and pyrolysis methods that are each potentially subject to interfering agents and could slightly overestimate the final result. Thus, the HILIC LC–MS/MS method reported here provided a quantitative approach to protein analysis accomplished through a single hydrolysis and processing run without the need for derivatization.

**Table 4:** Concentration of amino acids as measured by LC–MS/MS with isotope dilution three different hydrolysis methods

Analyte	H_2_O_2_ + 6 M HCl + β-ME	6 M HCl + β-ME	4 M MetS + 0.2% tryptamine	Reference*
Ala	1.6 ± 0.13	1.54 ± 0.08	1.53 ± 0.05	2.28 ± 0.16
Arg	2.86 ± 0.07	2.64 ± 0.08	2.85 ± 0.24	3.72 ± 0.31
Asx	5.2 ± 0.24	5.65 ± 0.38	5.77 ± 0.46	6 ± 1.2
Glx	7.8 ± 0.05	8.04 ± 0.76	8.5 ± 0.15	10.2 ± 1.4
Gly	1.69 ± 0.13	1.78 ± 0.01	1.82 ± 0.04	2.22 ± 0.15
His	1.25 ± 0.08	1.55 ± 0.1	1.72 ± 0.08	1.22 ± 0.09
Ile	2.59 ± 0.14	2.62 ± 0.22	2.37 ± 0.11	2.31 ± 0.23
Leu	4.62 ± 0.15	4.56 ± 0.49	5.13 ± 0.12	4.03 ± 0.42
Lys	3.49 ± 0.12	3.69 ± 0.2	4.03 ± 0.03	3.2 ± 0.25
Phe	2.22 ± 0.05	2.93 ± 0.06	3.21 ± 0.17	2.54 ± 0.13
Pro	2.73 ± 0.1	3.05 ± 0.08	3.22 ± 0.12	2.71 ± 0.23
Ser	3.03 ± 0.23	3.09 ± 0.24	2.92 ± 0.2	2.69 ± 0.32
Thr	1.97 ± 0.06	2.52 ± 0.18	2.33 ± 0.11	2.02 ± 0.11
Val	2.51 ± 0.1	2.41 ± 0.11	2.72 ± 0.13	2.45 ± 0.41
Tyr	0 ± 0	2.55 ± 0.24	3.13 ± 0.33	1.76 ± 0.43
Trp	0 ± 0	0 ± 0	0 ± 0	0.66 ± 0.14
Cys**	1.14 ± 0.04	0.81 ± 0.05	1.02 ± 0.13	0.74 ± 0.15
Met**	0.97 ± 0.07	0.74 ± 0.04	0.91 ± 0.07	0.69 ± 0.13
Total protein	48.04 ± 0.99	50.17 ± 0.89	53.19 ± 1.47	53.24 ± 0.36***

Values are expressed as mg/100 mg biomass, amino acids represented by three letter abbreviations

*Values were obtained from NIST certificate of analysis for standard reference material^®^ 3234. Variability is expressed as standard error of mean (*n* = 3)

**Cysteine was measured as cysteic acid in hydrolysis that involved prior oxidation and as cystine for the other two methods. Similarly, methionine was measured as methionine suloxide upon oxidation and as methionine in the two methods that did not involve prior oxidation

***Total protein reported by NIST used a combination of Kjeldahl, thermal conductivity and pyrolysis methods [48]. Summation of amino acids from NIST reference gives 51.44 mg/100 mg biomass

## Conclusions

**Fig. 4:**
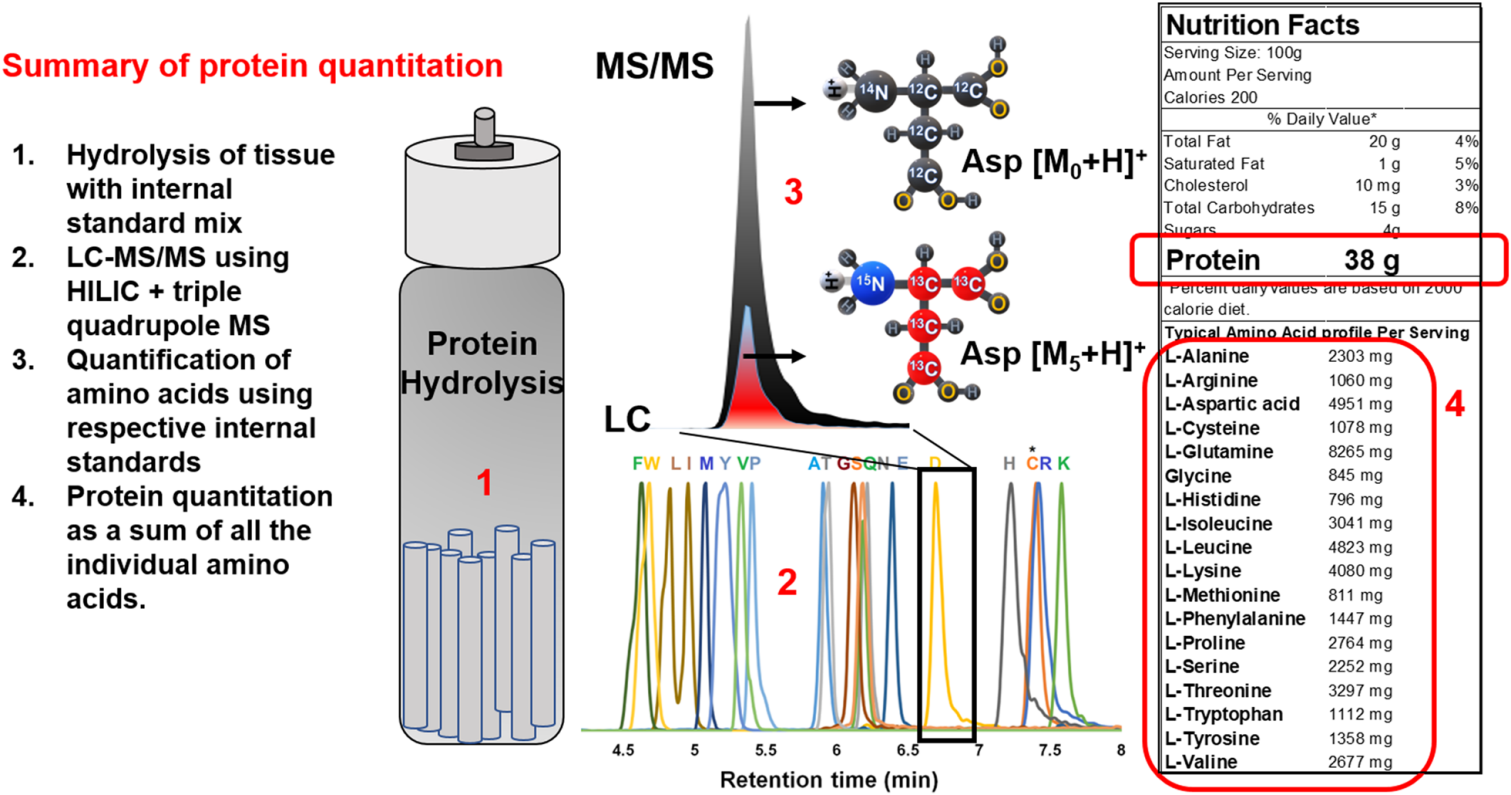
Graphical summary of protein quantitation using amino acid analysis via HILIC LC-MS/MS

**Fig. 5:**
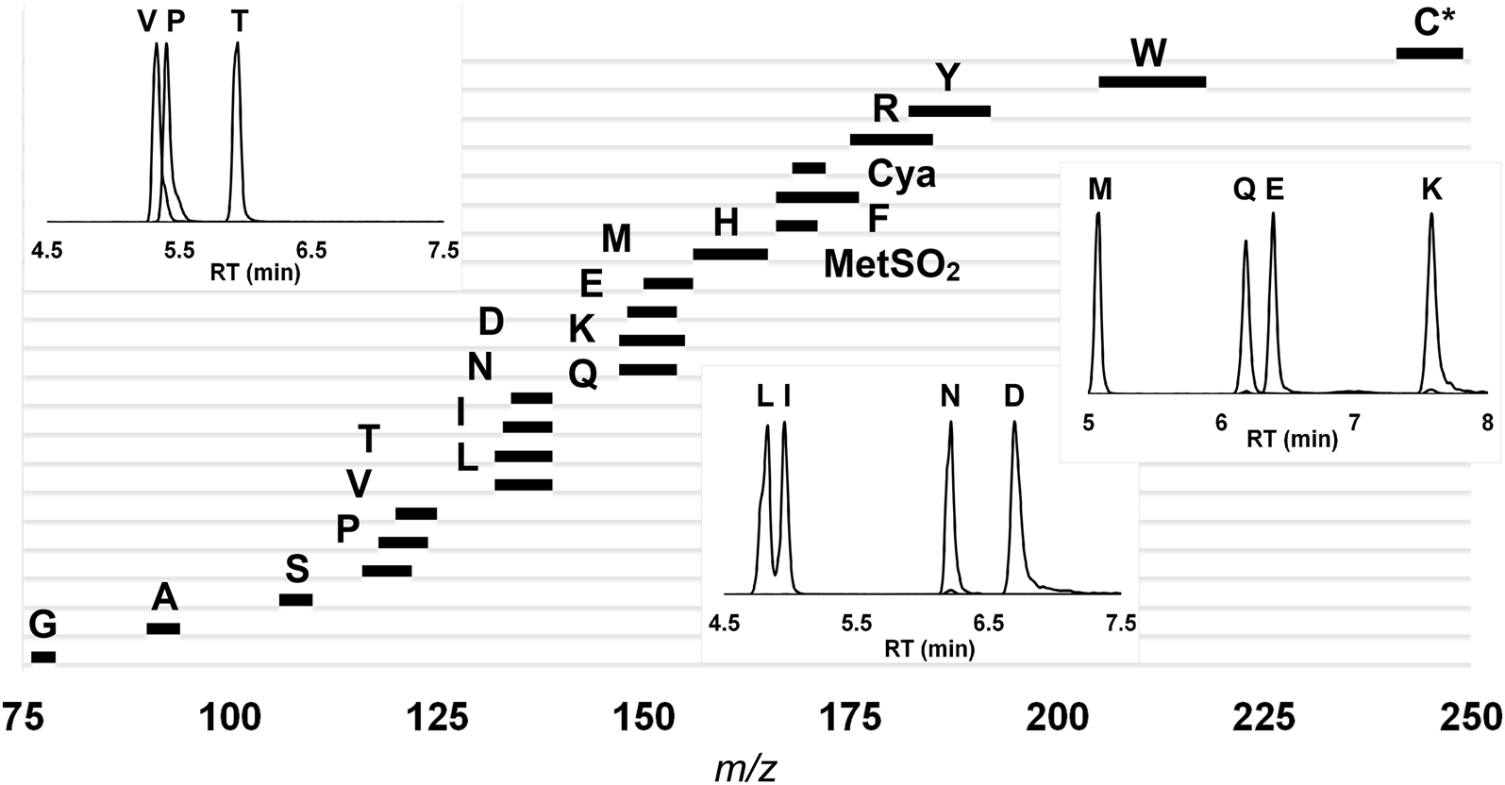
Overlap in mass ranges, M_0_ − M_n,_ where n is the maximum number of ^13^C and ^15^N isotopologues possible for each amino acid. The inset figures indicate separation of amino acids with overlapping masses that can be resolved by HILIC and used to quantify isotopologues. *Cysteine was measured as cystine

We developed a combined approach for amino acid compositional analysis and protein concentration determination using HILIC LC-MS/MS with isotope dilution-based quantitation. All twenty amino acids were resolved and quantified using a commercially available mix of labeled amino acids resulting in quantitative determination of protein levels. The method provides a robust and efficient strategy that could be extended to accommodate the detection of non-proteinogenic and modified amino acids [37–39] and can be used for semi-high throughput protein analysis without the need for derivatization after hydrolysis. A graphical summary of the method is presented in Fig. 4. The methane sulfonic acid method of hydrolysis resulted in the highest detected level of labile amino acids; however, since the labile amino acids are present at low levels, all hydrolysis methods resulted in accurate quantification when isotope dilution was employed. HILIC separates most amino acids such that potentially all overlapping isotopologues are resolved (Fig. 5). Alternatively, for combined protein concentration determination and quantification of isotope labeling at the intact protein level, analysis of isotopic fine structure could also be utilized by combining HILIC LC-MS/MS with high resolution mass spectrometry (HRMS) in order to resolve isotope-labeled analytes from isotope-labeled standards using distinct isotopes [59, 60].

## Additional files

**Additional file 1: Table S1.** Multiple reaction monitoring parameters for amino acids and their respective internal standards on a 6500 QTRAP LC–MS/MS.

**Additional file 2: Figure S1.** Amino acid quantitation from BSA hydrolyzed using seven different approaches from the literature [21, 56–58, 61]. Peak areas were log_10_ transformed for relative comparison. Methods included pre-oxidation with either H_2_O_2_ or performic acid, β-mercaptoethanol as a reducing agent, and presence or absence of phenol as an antioxidant when 6 M HCl was used for vapor-phase hydrolysis. In addition, a liquid-phase hydrolysis strategy using 4 M methane sulfonic acid with 0.2% tryptamine was also tested. Labile amino acids including: cysteine, methionine, tryptophan and tyrosine are presented with standard errors from duplicate preliminary experiments.

**Additional file 3: Fig.** **2.** Standard curves generated using a serial dilution (0.1–1000 pmol) of amino acid mixture spiked with ^13^C, ^15^N labeled internal standard mix. Ratios of peak areas (analyte vs internal standard) were plotted again ratios of concentrations of amino acids relative to internal standards. *Cysteine was detected as cystine.

## Data Availability

All data generated or analyzed during this study are included in this published article and its supplementary information files.
