# Supplementary material for: Accurate and efficient amino acid analysis for protein quantification using hydrophilic interaction chromatography coupled tandem mass spectrometry
Source: Plant Methods. 2019 May 11;15:46. doi: 10.1186/s13007-019-0430-z (PMC6511150; doi:10.1186/s13007-019-0430-z)
Supplement: Supplementary file 1 — Additional file 1: Table S1. Multiple reaction monitoring parameters for amino acids and their respective internal standards on a 6500 QTRAP LC–MS/MS. [file 13007_2019_430_MOESM1_ESM.docx]

**Table S1.** Multiple reaction monitoring parameters for amino acids and their respective internal standards on QTRAP 6500 LC-MS/MS.

| **Analyte / Internal standard** | **Q1** | **Q3** | **Dwell**  **time (ms)** | **CE** | **CXE** | **DP** |
| --- | --- | --- | --- | --- | --- | --- |
| Alanine | 90 | 44 | 7 | 16 | 43 | 8 |
| ^13^C_3_,^15^N Alanine | 94 | 47 | 7 | 16 | 43 | 8 |
| Arginine | 175 | 70 | 7 | 41 | 27 | 8 |
| ^13^C_6_,^15^N_4_ Arginine | 185 | 75 | 7 | 41 | 27 | 8 |
| Asparagine | 133 | 74 | 7 | 31 | 27 | 10 |
| ^13^C_4_,^15^N_2_ Asparagine | 139 | 77 | 7 | 31 | 27 | 10 |
| Aspartate | 134 | 74 | 7 | 36 | 17 | 10 |
| ^13^C_4_,^15^N Aspartate | 139 | 77 | 7 | 36 | 17 | 10 |
| Cysteic acid* | 167.9 | 80.8 | 7 | -60 | -26 | -9 |
| ^13^C_3_,^15^N Cysteic acid | 171.9 | 80.8 | 7 | -60 | -26 | -9 |
| Cysteine | 122 | 59 | 7 | 15 | 29 | 8 |
| ^13^C_3_,^15^N Cysteine | 126 | 61 | 7 | 15 | 29 | 8 |
| Cystine | 241.2 | 152.1 | 7 | 25 | 19 | 10 |
| ^13^C_6_,^15^N_2_ Cystine | 249.2 | 156.1 | 7 | 25 | 19 | 10 |
| Glutamate | 148 | 84 | 7 | 66 | 21 | 10 |
| ^13^C_5_,^15^N Glutamate | 154 | 89 | 7 | 66 | 21 | 10 |
| Glutamine | 147 | 56 | 7 | 60 | 35 | 8 |
| ^13^C_5_.^15^N_2_ Glutamine | 154 | 89 | 7 | 60 | 23 | 10 |
| Glycine | 76 | 30 | 7 | 6 | 19 | 14 |
| ^13^C_2_,^15^N Glycine | 79 | 32 | 7 | 6 | 19 | 14 |
| Histidine | 156 | 110 | 7 | 31 | 19 | 14 |
| ^13^C_9_,^15^N_3_ Histidine | 165 | 118 | 7 | 31 | 19 | 14 |
| Isoleucine | 132 | 69 | 7 | 8 | 23 | 8 |
| ^13^C_6_,^15^N Isoleucine | 139 | 74 | 7 | 8 | 23 | 8 |
| Leucine | 132 | 86 | 7 | 26 | 13 | 8 |
| ^13^C_6_,^15^N Leucine | 139 | 92 | 7 | 26 | 13 | 8 |
| Lysine | 147 | 84 | 7 | 6 | 21 | 10 |
| ^13^C_6_,^15^N_2_ Lysine | 155 | 90 | 7 | 6 | 21 | 10 |
| Methionine | 150 | 104 | 7 | 16 | 11 | 6 |
| ^13^C_5_,^15^N Methionine | 156 | 109 | 7 | 16 | 11 | 6 |
| Methionine sulfoxide | 166.11 | 56.036 | 7 | 27 | 33 | 8 |
| ^13^C_5_,^15^N Methionine sulfoxide | 171.11 | 60.036 | 7 | 27 | 33 | 8 |
| Phenylalanine | 166 | 120 | 7 | 21 | 17 | 16 |
| ^13^C_9_,^15^N Phenylalanine | 176 | 129 | 7 | 21 | 17 | 16 |
| Proline | 116 | 70 | 7 | 36 | 21 | 8 |
| ^13^C_5_,^15^N Proline | 122 | 75 | 7 | 36 | 21 | 8 |
| Serine | 106 | 60 | 7 | 16 | 15 | 8 |
| ^13^C_3_,^15^N Serine | 110 | 63 | 7 | 16 | 15 | 8 |
| Threonine | 120 | 74 | 7 | 16 | 15 | 10 |
| ^13^C_4_,^15^N Threonine | 125 | 78 | 7 | 16 | 15 | 10 |
| Tryptophan | 205 | 146 | 7 | 11 | 23 | 12 |
| ^13^C_11_,^15^N_2_ Tryptophan | 218 | 156 | 7 | 11 | 23 | 12 |
| Tyrosine | 182 | 136 | 7 | 26 | 17 | 10 |
| ^13^C_9_,^15^N Tyrosine | 192 | 145 | 7 | 26 | 17 | 10 |
| Valine | 118 | 72 | 7 | 26 | 15 | 10 |
| ^13^C_5_,^15^N Valine | 124 | 77 | 7 | 26 | 15 | 10 |
