# Supplementary figures and images for: Accurate and efficient amino acid analysis for protein quantification using hydrophilic interaction chromatography coupled tandem mass spectrometry
Source: Plant Methods. 2019 May 11;15:46. doi: 10.1186/s13007-019-0430-z (PMC6511150; doi:10.1186/s13007-019-0430-z)

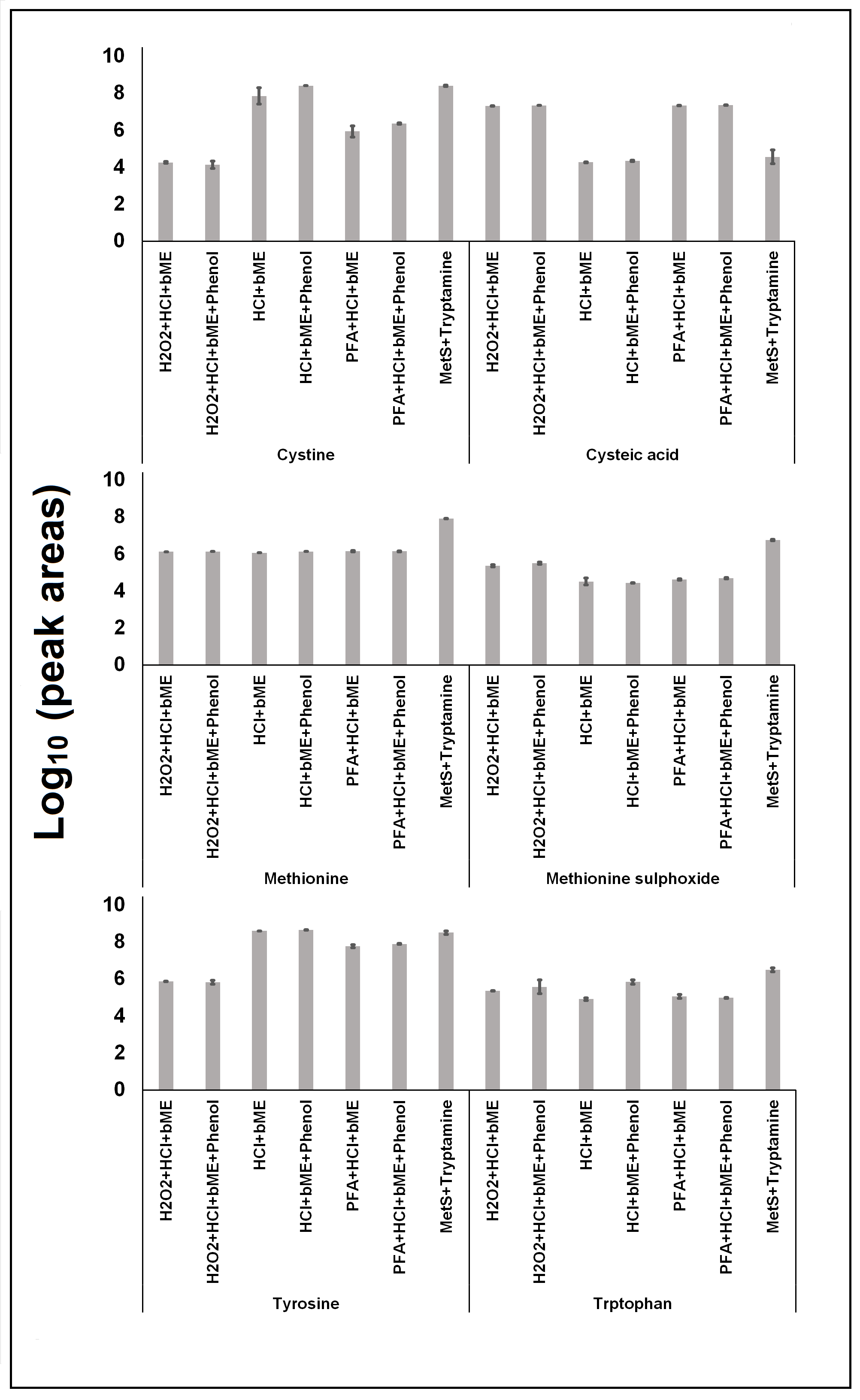

Supplement: Supplementary file 2 — Additional file 2: Figure S1. Amino acid quantitation from BSA hydrolyzed using seven different approaches from the literature [21, 56–58, 61]. Peak areas were log10 transformed for relative comparison. Methods included pre-oxidation with either H2O2 or performic acid, β-mercaptoethanol as a reducing agent, and presence or absence of phenol as an antioxidant when 6 M HCl was used for vapor-phase hydrolysis. In addition, a liquid-phase hydrolysis strategy using 4 M methane sulfonic acid with 0.2% tryptamine was also tested. Labile amino acids including: cysteine, methionine, tryptophan and tyrosine are presented with standard errors from duplicate preliminary experiments. [file 13007_2019_430_MOESM2_ESM.tif]

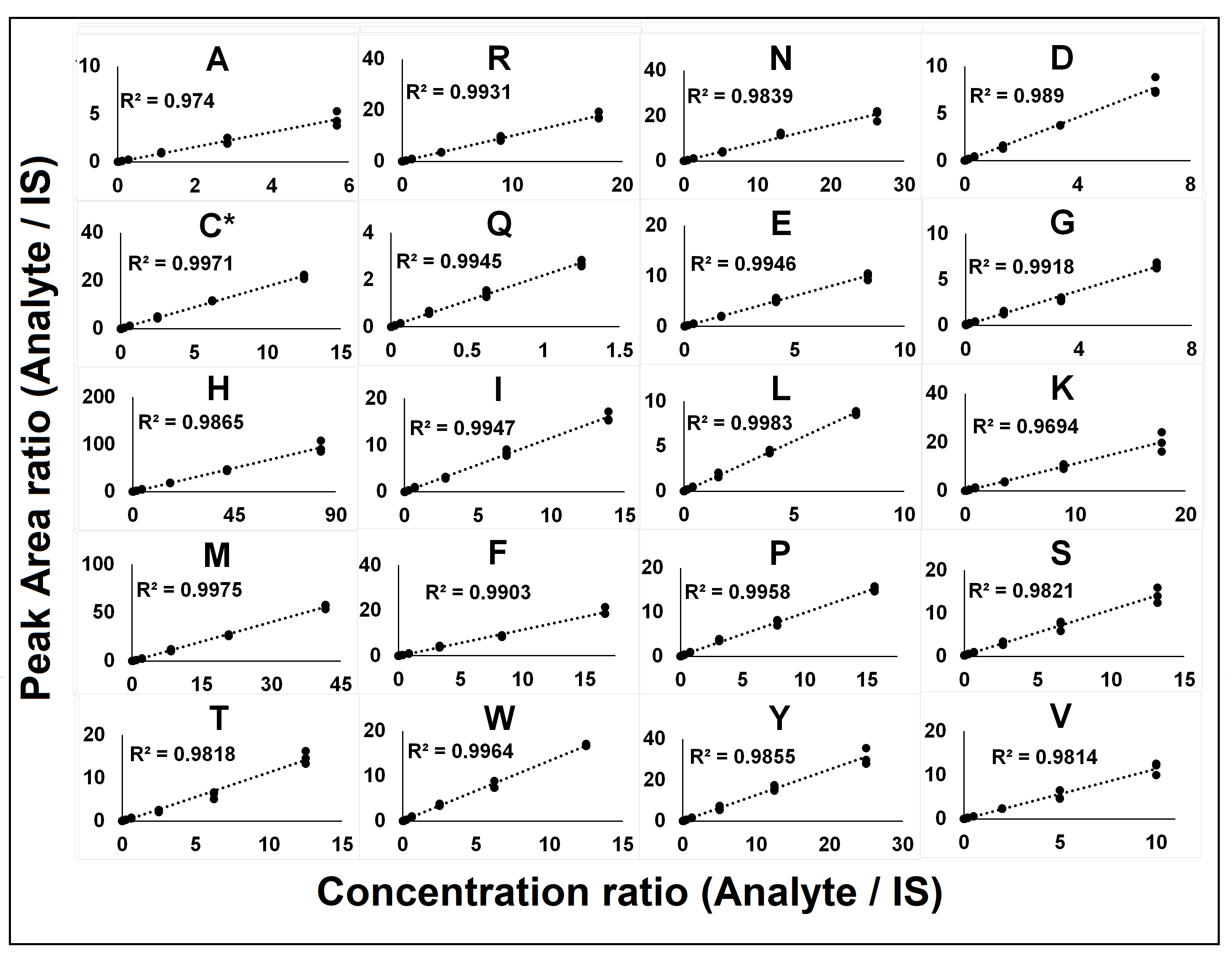

Supplement: Supplementary file 3 — Additional file 3: Fig. 2. Standard curves generated using a serial dilution (0.1–1000 pmol) of amino acid mixture spiked with 13C, 15N labeled internal standard mix. Ratios of peak areas (analyte vs internal standard) were plotted again ratios of concentrations of amino acids relative to internal standards. *Cysteine was detected as cystine. [file 13007_2019_430_MOESM3_ESM.tif]
